# Bacterial contaminants from frozen puff pastry production process and their growth inhibition by antimicrobial substances from lactic acid bacteria

**DOI:** 10.1002/fsn3.413

**Published:** 2016-08-05

**Authors:** Kittaporn Rumjuankiat, Suttipun Keawsompong, Sunee Nitisinprasert

**Affiliations:** ^1^ Specialized Research Unit: Prebiotics and Probiotics for HealthFaculty of Agro‐IndustryDepartment of BiotechnologyKasetsart UniversityBangkokThailand; ^2^ Center for Advanced Studies for Agriculture and FoodKasetsart University Institute for Advanced StudiesKasetsart UniversityBangkokThailand

**Keywords:** Antimicrobial substance, bacterial contaminants, disinfectant solution, frozen puff pastry, lactic acid bacteria

## Abstract

Seventy‐five bacterial contaminants which still persisted to cleaning system from three puff pastry production lines (dough forming, layer and filling forming, and shock freezing) were identified using 16S rDNA as seven genera of *Bacillus*,* Corynebacterium*,* Dermacoccus*,* Enterobacter*,* Klebsiella, Pseudomonas*, and *Staphylococcus* with detection frequencies of 24.00, 2.66, 1.33, 37.33, 1.33, 2.66, and 30.66, respectively. Seventeen species were discovered while only 11 species *Bacillus cereus, B. subtilis, B. pumilus, Corynebacterium striatum*,* Dermacoccus barathri*,* Enterobacter asburiae, Staphylococcus kloosii, S. haemolyticus, S. hominis, S. warneri*, and *S. aureus* were detected at the end of production. Based on their abundance, the highest abundance of *E. asburiae* could be used as a biomarker for product quality. While a low abundance of the mesophile pathogen *C. striatum*, which causes respiratory and nervous infection and appeared only at the shock freezing step was firstly reported for its detection in bakery product. Six antimicrobial substances (AMSs) from lactic acid bacteria, FF1‐4, FF1‐7, PFUR‐242, PFUR‐255, PP‐174, and nisin A were tested for their inhibition activities against the contaminants. The three most effective were FF1‐7, PP‐174, and nisin A exhibiting wide inhibition spectra of 88.00%, 85.33%, and 86.66%, respectively. The potential of a disinfectant solution containing 800 AU/ml of PP‐174 and nisin A against the most resistant strains of *Enterobacter*,* Staphylococcus*,* Bacillus* and *Klebsiella* was determined on artificially contaminated conveyor belt coupons at 0, 4, 8, 12, and 16 hr. The survival levels of the test strains were below 1 log CFU/coupon at 0 hr. The results suggested that a combined solution of PP‐174 and nisin A may be beneficial as a sanitizer to inhibit bacterial contaminants in the frozen puff pastry industry.

## 1 Introduction

Foodborne and pathogenic bacterial contamination of foodstuffs is a global issue as a serious hazard to human health, and also results in high spoilage losses (Muhialdin et al., 2012). *Bacillus cereus*,* Escherichia coli*, and *Staphylococcus aureus* are pathogens that have been responsible for the outbreaks of foodborne disease often found in several foodstuffs including bakery products (Lindqvist et al., 2001; Matarante, Baruzzi, Cocconcelli, & Morea, 2004; Muhialdin et al., 2012). *B. cereus* associated with gastrointestinal disorders can cause two types of food poisoning (emetic and diarrheal type). Additionally, it is also an opportunistic human pathogen associated with systemic infections such as necrotizing infections, endocarditis, sepsis, liver abscess, pneumonia, or meningitis (Ramarao & Sanchis, 2013). Shiga toxin (Stx)‐producing *Escherichia coli* (STEC) can cause diarrheal illness, hemolytic uremic syndrome (HUS) and even death. Especially, *E. coli* O157:H7 is the most commonly reported in outbreaks and its toxin typically affects children, the elderly, and immunocompromised patients (Khosravi, Khaghani, Sheikh, Zadeh, & Shamsizadeh, 2016; King et al., 2014; Lupindu et al., 2014). While *S. aureus* is an important foodborne pathogenic bacteria that can produce various enterotoxins. Staphylococcal enterotoxins can cause diarrhea, vomiting, and abdominal pain. Furthermore, the symptoms of headaches, prostration, and low blood pressure have been reported in the most severe cases (Argudín, Mendoza, & Rodicio, 2010; Hennekinne, De Buyser, & Dragacci, 2012). Furthermore, *Enterobacter hormaechei* detected in humans is also considered as a human disease (Wenger et al., 1997). These pathogens can originate mainly from raw materials and equipment (Bailey & von Holy, 1993; Rosenkvist & Hansen, 1995). Production process in food industry consisting of trimming, cutting, washing, rinsing, dewatering, or packaging are considered to be the primary source of cross‐contamination (Srey, Jahid, & Ha, 2013). Occasionally, microbial control strategies are inefficient to completely eliminate microorganisms. Therefore, there are many foodborne illness outbreaks have been reported by poor hygiene such as ineffective hand washing or inadequate temperature controls of food (Lambrechts, Human, Doughari, & Lues, 2014). These wrong manipulation and food nature can trigger pathogenic contamination. Nowadays, convenient food like bakery products has become more popular among Thai people reflecting the change of their lifestyle (Wongsuttichote & Nitisinprasert, 2009). Puff pastry is a kind of bakery products which is popular nowadays due to varieties of taste and ease of consumption. Its production rate had gradually increased for 5–12% during the past 3 years. Puff pastry has a laminated structure of baked layers of dough separated by thin layers of fat with varieties of fillings causing the short shelf life (Hay, 1993). Kotzekidou (2013) reported the contamination of foodborne pathogen in frozen pastries including *Bacillus cereus*,* Salmonella* spp., *Escherichia coli* O157:H7, *Listeria monocytogenes*, and *Staphylococcus aureus* in raw material, cross‐contamination during preparation, and handling causing reduction of product shelf life. Biopreservation is another option for food safety to extend its shelf life (Stiles, 1996). Many reports have noted the success of lactic acid bacteria (LAB) for the growth inhibition of pathogens and foodborne contaminants (Muhialdin et al., 2012). LAB produces numerous antimicrobial substances (AMS) including organic acids, hydrogen peroxide, diacetyl, carbon dioxide, reuterin, and bacteriocins (Galvez, Burgos, Lopez, & Pulido, 2014), which may inhibit nonpathogens and pathogens associated with food and humans (Corsetti, Settanni, Braga, De Fatima Silva Lopes, & Suzzi, 2008). Among these, nisin is a lantibiotic antimicrobial peptide which is produced by *Lactococcus lactis*. Nisin exhibited bactericidal activity against foodborne bacteria including *Bacillus cereu*s, *Staphylococcus aureus*,* Listeria monocytogenes* and recognized as safe food preservative (Wiedemann et al., 2001). In addition, it is odorless, colorless, tasteless, and has a low toxicity (Tong, Ni, & Ling, 2014). Wongsuttichote and Nitisinprasert (2009) reported that the cell‐free supernatants (CFS) from *Lactobacillus plantarum* KUB‐KJ174 isolated from fermented rice noodles displayed spectrum inhibition activity of 100% against bacterial contaminants of 50 aerobic mesophiles, 10 *Bacillus cereus*, and 53 coliform tested but only 46% inhibition spectrum activity against 13 LAB isolated from bakery product namely “E’ Claire”. Partial purification by pH mediated cell adsorption–desorption providing an active AMS so called PP‐174 displayed 100% growth inhibition activities against microbial strains of aerobic mesophile bacteria T6.14, *B. cereus* B6.2, coliform C4.1, LAB L2.2, and yeast Y5.1. In our previous study, AMS producing LAB have been screened from various fermented food in Thailand. The cell‐free supernatants (CFS) namely FF1‐4, FF1‐7, PFUR‐242, and PFUR‐255 produced by four strains of KUB‐KJ174, KA‐FF1‐4, KA‐FF1‐7, KA‐PFUR‐242, and KA‐PFUR‐255, respectively, grown in MRS medium exerted high inhibition activities against *Staphylococcus aureus* TISTR 029. These LAB strains can be source of AMS used for biopreservatives in the future.

In Thailand, these products had a short shelf life of only 1–2 days. To maintain the fresh product, the puff pastry is maintained as a dough form at low temperature as a frozen product. They will be baked at high temperature before serving or distribution to the customer. All raw materials and equipments for three lines of dough‐forming, layer and filling‐forming and shock‐frozen process need to be aware for food safety. Normally, all equipments used for industrial puff pastry production are properly cleaned by disinfectant agents followed by water at the end. However, sanitation test by swab test of all equipments before usage showed the occurrence of some bacterial contaminants which could be resistant to the cleaning system. Therefore, this research aimed to identify the bacterial contaminants which persisted to the cleaning system and further investigate for potential AMS for their growth inhibition.

## 2 Materials and Methods

### 2.1 Bacterial contaminant sources and morphological analysis

All bacterial contaminants were collected from commercial bakery industry located in Bangkok, Thailand. The temperature during process could be about 25–37°C. They were obtained from sanitation test using swab test of all equipments before usage. Briefly, all machines and equipments were properly cleaned with detergents and water, and then left for overnight at the temperature of about 25–37°C to obtain the dry ones. Before the production started, sanitation test were performed by swab test on PCA (Merck, Germany). Each bacterial colony having different characters appear was subsequently purified on nutrient agar (NA) to obtain 8, 52, and 15 isolates from three production lines including the dough‐forming line (DL), the layer‐and‐filling line (LFL), and the shock‐frozen line (SL), respectively (Table 1). All culture isolates were propagated in Nutrient Broth medium (NB; Merck, Germany) under aerobic conditions by shaking at 200 rpm at 37°C overnight and determined for their morphologies by an optical microscope (Primo Star, Thornwood, NY, USA).

**Table 1:** The bacterial contaminants from the puff pastry production processes

Production line	Bacterial isolates
Dough forming	TPC‐T44, TPC‐T45, TPC‐T21, TPC‐T22, TPC‐T23, TPC‐T24, TPC‐T25, TPC‐Y4
Layer and filling forming	TPC‐Y1, TPC‐T42, TPC‐T43, TPC‐T9, TPC‐T10, TPC‐T31, TPC‐T32(S), TPC‐T32(B), TPC‐T56, TPC‐Y5, PC‐Y8, TPC‐T30, TPC‐T19, TPC‐T20, TPC‐T36, TPC‐T37, TPC‐T13, TPC‐T14(S), TPC‐T14(B), TPC‐T15(S), TPC‐T15(B), TPC‐T16(S), TPC‐T16(B), TPC‐T17(S), TPC‐T17(B), TPC‐T33, PC‐T6, PC‐T2, PC‐Y2, PC‐T12, PC‐T13, PC‐T14, PC‐T4, PC‐T7, PC‐T8, TPC‐T27, TPC‐T28, TPC‐T39, TPC‐T29, TPC‐T50(S), TPC‐T50(B), TPC‐T51, TPC‐T35, TPC‐T38, TPC‐T46, TPC‐T47, TPC‐Y6, TPC‐T57, TPC‐T53, PC‐T9, PC‐T10, TPC‐T52
Shock frozen	TPC‐T5, TPC‐T6, TPC‐T49, TPC‐T54, TPC‐T48, TPC‐Y10, TPC‐Y11, TPC‐T40, TPC‐T1, TPC‐T2, TPC‐T3(S), TPC‐T3(B), PC‐Y9, PC‐Y10, PC‐T11

### 2.2 Antimicrobial substances

Six AMS of nisin A (Nisaplin^®^, Aplin and Barret, UK), PP‐174 (partial purified AMS) produced by *L. plantarum* KUB‐KJ174 and four AMS of FF1‐4, FF1‐7, PFUR‐242 and PFUR‐255 in the form of cell‐free supernatant produced by four LAB strains of KUB‐KJ174, KA‐FF1‐4, KA‐FF1‐7, KA‐PFUR‐242, and KA‐PFUR‐255, respectively, grown in MRS medium at 37°C for 16–18 hr were studied. Nisin A was prepared by dissolving in 0.02 N HCl pH 2 to obtain a final concentration of 0.01 g/ml (10^4^ IU/ml) and then filtering through a 0.20 μm filter membrane (Sartorius Stedim Biotech GmbH, Goettingen, Germany) while PP‐174 was prepared as partial purification by pH adsorption and desorption according to the method of Wongsuttichote and Nitisinprasert (2009).

### 2.3 DNA extraction

Bacterial DNA was extracted from each overnight culture solution using the bacteria genomicPrep Mini Spin Kit (Illustra^™^, Buckinghamshire, UK) and performed according to the manufacturer's instructions. The extracted DNA was subjected to randomly amplified polymorphic DNA polymerase chain reaction (RAPD‐PCR) for biotype screening and 16S rDNA analysis.

### 2.4 RAPD‐PCR typing

The 10‐base pair primers of OPA3 (5′‐AGTCA GCCAC‐3′) and OPA11 (5′‐CAATCGCCGT‐3′) which were described by Sorokulova et al. (2003) and Baker, Crumley, and Eckdahl (2002), respectively, were used in this study. The reactions were prepared in a total volume of 25 μl containing 10–50 ng of genomic DNA, 0.4 μmol/L of each primer, 0.2 mmol/L of dNTP mix, 1.5U of Taq DNA polymerase (Fermentas, Waltham, Massachusetts, USA), and 10× PCR Buffer with MgCl_2_. The DNA was amplified using the following program: 94°C for 1 min and 45 cycles of 94°C for 1 min, 36°C for 1 min, 72˚C for 1 min, and a final extension of 72°C for 10 min. Amplified PCR products were verified using electrophoresis in 1.5% agarose gel (Mupid^®^ex, Tokyo, Japan) containing ethidium bromide according to the method of Devos and Gale (1992). The DNA marker used to determine the size of amplified fragments was O'GeneRuler^™^ 1 kb DNA Ladder (Fermentas, Waltham, Massachusetts, USA). Each DNA fragment was visualized using a UV transilluminator and later photographed. The clearest and most reproducible bands were chosen for determination of their presence and absence in each isolate. Faint bands which could not be systematically visualized were not taken into account.

### 2.5 Cluster analysis

The observed bands in the gels were evaluated based on the presence (coded 1) or absence (coded 0) of polymorphic fragments for RAPD products. Cluster analysis was performed with the NTSYS‐pc (version 2.10e) software, which is a numerical taxonomy and multivariate analysis software package (Rohlf, 2000). The similarity among digitized profiles was calculated using the SAHN. The dendrogram was constructed using an unweighted pair group method with arithmetic (UPGMA) cluster analysis.

### 2.6 Analysis of 16S rDNA nucleotide sequence

Bacterial DNA was extracted from each culture solution using the bacteria genomic Prep Mini Spin Kit (Illustra^™^, Buckinghamshire, UK), performed according to the manufacturer's instructions. Genomic DNA from each bacterial isolate was used as a template to amplify 16S rDNA gene fragments, using polymerase chain reaction (PCR) technique with the universal primers BSF8/20 (5′‐AGAGTTTGATCCTGGCTCAG‐3′) and REVB (5′‐GGTTACCTTGTTACGACTT‐3′) (Kanokratana, Chanapan, Pootanakit, & Eurwilaichitr, 2004). Each DNA amplification was performed in a 50 μl PCR reaction containing 0.05 units *Taq* DNA Polymerase, 10× PCR Buffer, 0.1 mmol/L dNTP mix (Fermentas, Waltham, Massachusetts, USA), 0.4 μmol/L of each primer, and 10–50 ng DNA template. The reaction was performed in a thermal cycler (Biometra, Germany) using the following program: 94°C for 1 min and 45 cycles of 94°C for 1 min, 36°C for 1 min, 72°C for 1 min, and a final extension of 72°C for 10 min. PCR products were verified using electrophoresis on 1.5% agarose (Mupid^®^ex, Tokyo, Japan) gel containing ethidium bromide. The obtained PCR products were purified using a B.E.Z.N.A.^™^ gel extraction kit (Omega Bio‐Tek, Norcross, GA, USA), and further cloned into pTZ57R/T vector (InsTAclone^™^ PCR cloning Kit, Fermentas, Waltham, Massachusetts, USA) using the *E. coli* strain DH5α as a host cell, and screened using blue/white colonies. The recombinant plasmids were purified using QIAprep^®^ Spin miniprep kit (QIAGEN, Hilden, Germany), and sequenced by the 1st Based Company (Malaysia). The sequence identity of each sample was determined using BLAST comparisons to the GenBank database.

### 2.7 Determination of the detection frequency

The detection frequencies of the 75 bacterial contaminant isolates collected from the three production lines were determined. They were expressed as the percentage of isolates belonging to each genus of the total isolates, and the percentage belonging to each species of the total isolates for each production line.

### 2.8 Determination of inhibition spectra

All bacterial contaminants were used as target isolates to determine their inhibition activities according to the modified method of Ennahar, Asou, Zendo, Sonomoto, and Ishizaki (2001). In brief, 10 μl of each AMS sample exhibiting about 200 AU/ml against the target strain *Staphylococcus aureus* TISTR 029 was spotted onto the surface of an nutrient agar plate which was overlaid with 5 ml of 1.0% soft agar (Difco Laboratories, USA), seeded with 20 μl of each freshly grown target strain (OD_600_ = 0.1). After overnight incubation at 37°C, the inhibition zones on the bacterial lawn were determined. The isolates showing a clear zone by each AMS were defined as positive results. The inhibition spectrum was calculated using the equation:

$$\text{Inhibition spectrum}=N_{\mathrm{inhibition}}\times 100/N_{\mathrm{total}}$$

where *N*_inhibition_ is the number of bacterial contaminant isolates inhibited by each AMS and *N*_total_ is the total number of bacterial contaminant isolates tested.

### 2.9 Artificial contamination of conveyor belt coupon and determination of antimicrobial substance efficiency

Conveyor belt coupons (size 2 × 2 cm^2^) were used as a model and prepared according to the modified method described by Phongphakdee and Nitisinprasert (2015). Each conveyor belt sheet was cleaned with distilled water and autoclaved at 121°C for 15 min. Twenty‐one representative bacterial contaminants resistant to either PP‐174 or nisin A (Table 2) were mixed and used to perform the contaminant coupons. One milliliter of each bacterial isolates propagated in NB medium by shaking at 200 rpm at 37°C for 16–18 hr and adjusted to an optical density of 0.1 at 600 nm (approximately 10^7^ CFU/ml) was mixed to obtain the target culture mixture solution. Ten microliters of culture mixture solution were inoculated to coupon and dried in a laminar flow for 1 hr. Each conveyor belt coupon containing dried cell of target isolates was treated with PP‐174, nisin A alone, and a combination of PP‐174 and nisin A (1:1), adjusted to the antimicrobial activity of 1600 AU/ml against the growth of *B. cereus* JCM 2152^T^. The 10 μl of each AMS solution was transferred to the contaminating position on each artificial contaminated coupons and incubated for 0, 4, 8, 12, and 16 hr at room temperature. The 0.85% NaCl was used as a control. Afterward, the coupons were transferred to conical centrifuge tubes containing 30 ml of steriled normal saline (0.85% w/v NaCl) and vortexed at the maximum speed for 1 min for cell dispersion. Aliquots 1 ml of sample were 10‐fold serially diluted with 9 ml of normal saline solution and analyzed by standard plate count method using NA medium at 37°C for 24 hr to determine its survival cell/coupon.

**Table 2:** The bacterial contaminants from the two production processes, dough forming and layer‐filling forming, not inhibited by either PP‐174 or nisin A

Production line	Resistant isolates	Bacterial species
Dough forming	TPC‐T23	*E. asburiae*
	TPC‐T45, PC‐T8	*S. warneri*
Layer and filling forming	TPC‐T31, TPC‐T56, TPC‐T20 TPC‐T13, TPC‐T14(B), TPC‐T27, TPC‐T50(S), TPC‐T46, PC‐T10	*E. asburiae*
	TPC‐T42, TPC‐T36, PC‐T9	*E. cloacae* subsp. *cloacae*
	TPC‐T30	*E. homaechei*
	TPC‐T52	*K. oxytoca*
	TPC‐Y6	*S. epidermidis*
	TPC‐T9, TPC‐T15(S), TPC‐T39	*S. kloosii*

### 2.10 Statistical analysis

A one‐way ANOVA was performed to determine the standard deviations and statistical significance of data at the 95% confidence interval (*p *<* *.05). All treatments were performed in duplicate. All analyzes were carried out using the SPSS statistical package version 16.0.

## 3 Results

### 3.1 Morphological analysis of the bacterial contaminants

Seventy‐five bacterial contaminants from three processes of puff pastry line production consisting of dough‐forming line (DL), the layer‐forming‐and‐filling line (LFL), and the shock‐frozen line (SL) were observed for their morphologies. These bacterial contaminants were classified into three groups including (1) gram‐positive bacteria in rod shape, (2) gram‐positive bacteria in coccus shape, and (3) gram‐ negative bacteria in rod shape as shown in Table 3.

**Table 3:** Morphological characteristic of contaminant bacteria

Process	Samples	Colony color	Gram stain	Shape
Dough forming	(i) TPC‐T44	White	+	Rod
	(ii) TPC‐T45, TPC‐T21, TPC‐T25, TPC‐Y4	White	+	Coccus
	(iii) TPC‐T22, TPC‐T23, TPC‐T24	White	−	Rod
Layer and filling forming	(i) TPC‐T15(B), TPC‐T16(B), TPC‐T32(B),TPC‐T37, PC‐T2, TPC‐T57, PC‐Y8,TPC‐T47, TPC‐Y5, TPC‐T16(S), TPC‐ T17(S),TPC‐T50(B)	White	+	Rod
	(ii) TPC‐T30, TPC‐T36, TPC‐T42, TPC‐T43,TPC‐T10, TPC‐T27, TPC‐T20, PC‐T9, TPC‐T31, TPC‐T35, TPC‐T46, PC‐T6, TPC‐T50(S), TPC‐T51, TPC‐T56, PC‐T10, PC‐T12, PC‐T14, PC‐T7, TPC‐T32(S), TPC‐T19, PC‐T4, TPC‐T17(B), TPC‐T14(B), TPC‐T13, TPC‐T52, TPC‐T38,	White	−	Rod
	(iii) TPC‐T28, PC‐Y2, TPC‐T29, PC‐T8, TPC‐T14(S), TPC‐Y6, TPC‐T15(S), TPC‐T39, TPC‐Y1, TPC‐T53, TPC‐T9, TPC‐T33, PC‐T13	White	+	Coccus
Shock frozen	(i) TPC‐T5, TPC‐T6, TPC‐Y10, TPC‐T1, PC‐T11, TPC‐T3(S), TPC‐T3(B)	White	+	Rod
	(ii) TPC‐T49, TPC‐T54, TPC‐T48, TPC‐Y11, TPC‐T40, PC‐Y10, PC‐Y9	White	+	Coccus
	(iii) TPC‐T2	White	−	Rod

### 3.2 Grouping and identification of bacterial contaminants from puff pastry production

**Figure 1:**
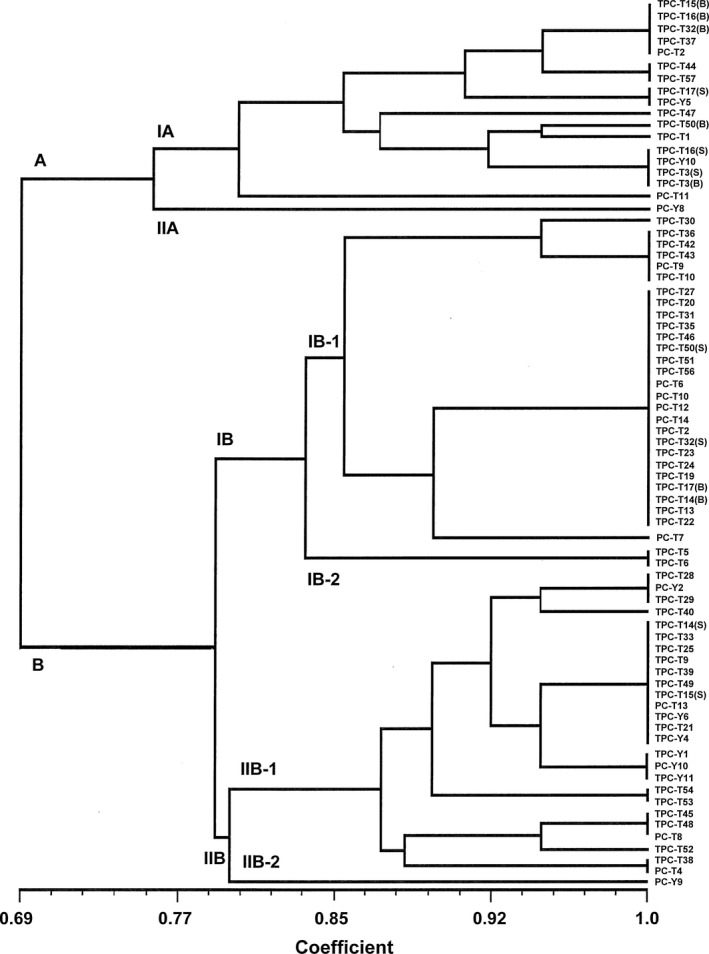
Phylogenetic tree constructed using the Unweighted Pair Group Method with Arithmetic Averages (UPGMA). Banding RAPD data from 75 bacterial contaminants were analyzed using NTSYSpc version2.10e software and produced two groups (A and B)

All 75 isolates collected from sanitation test in the puff pastry production process were first grouped by RAPD‐PCR using each of the two RAPD primers individually. However, only the OPA11 primer was found to be appropriate since it produced the clearer and more reproducible bands with all 75 test isolates and was, therefore, chosen for the RAPD‐PCR assays to analyze the bacterial contaminants. In total, 23 different RAPD‐PCR patterns were produced and resulted in 1 to 210 bands in the range 600–10,000 bp. The isolates representing each RAPD‐PCR pattern were identified using 16S rDNA sequence analysis to determine the species (Table 4). Phylogenetic trees were constructed using cluster analysis and resulted in two clusters named A and B as shown in Figure 1. Most gram‐positive, spore‐forming, aerobic rods belonged to cluster A which was divided into two subclusters of IA and IIA composed of different species of *Bacillus* and *B. thuringgiensis*, respectively, by 75% similarity. Cluster B was divided into two subclusters of IB and IIB by 79% similarity. Subcluster IB mainly consisted of gram‐positive rods and lactose‐fermenting, aerobic, gram‐negative rods of *Corynebacterium striatum* and *Enterobacter homaechei*,* E. cloacae* and of *E. asburiae*, respectively. Subcluster IIB contained two subclusters of IIB‐1 and IIB‐2. Subcluster IIB‐1 consisted of lactose‐fermenting, aerobic, gram‐negative rods (*Klebsiella oxytoca*), nonlactose fermenting ones (*Pseudomonas stutzeri*) and gram‐positive, aerobic cocci (*Staphylococcus epidermidis, S. aureus, S. kloosii*,* S. haemolyticus, S. hominis, S. warneri*). *Dermacoccus barathii* was the only gram‐positive coccus detected in subcluster IIB‐2.

**Table 4:** Identification of bacterial isolates using 16S rDNA analysis

RAPD pattern	Bacterial isolates	Bacterial species related	Similarity (%)	Abundance (%)
1	TPC‐T15(B), TPC‐T16(B), TPC‐T32(B), TPC‐T37, PC‐T2	*B. cereus* strain HS‐MP13	99	6.66
2	TPC‐T44, TPC‐T57	*B. cereus* strain SQT	99	2.66
3	TPC‐T17(S), TPC‐Y5	*B. cereus* strain ATCC 10987	99	2.66
4	TPC‐T47	*B. pumilus* strain PRE 14	99	1.33
5	TPC‐T50(B)	*B. cereus* strain DZ‐h	99	1.33
6	TPC‐T1	*B. cereus* strain DZ‐h	99	1.33
7	(i) TPC‐T16(S)	(i) *B. cereus* strain A168	99	1.33
	(ii) TPC‐Y10, TPC‐T3(S)	(ii) *B. subtilis* strain L4		2.66
	(iii) TPC‐T3(B)	(iii) *B. subtilis* strain 951NA4		1.33
8	PC‐T11	*B. pumilus* strain PRE 14	100	1.33
9	PC‐Y8	*B. thuringgiensis* strain DAB‐Bt4	91	1.33
10	TPC‐T30	*E. homaechei* strain PSB30	86	1.33
11	TPC‐T36, TPC‐T42, TPC‐T43, PC‐T9, TPC‐T10	*E. cloacae* subsp. *cloacae* NTCC 9394	99	6.66
12	TPC‐T27, TPC‐T20, TPC‐T31, TPC‐T35, TPC‐T46, TPC‐T50(S), TPC‐T51, TPC‐T56, PC‐T6, PC‐T10, PC‐T12, PC‐T14, TPC‐T2, TPC‐T32(S), TPC‐T23, TPC‐T24, TPC‐T19, TPC‐T17(B), TPC‐T14(B), TPC‐T13, TPC‐T22	*E. asburiae* strain E53	99	28.00
13	PC‐T7	*E. asburiae* strain E53	99	1.33
14	TPC‐T5, TPC‐T6	*C. striatum* strain Minnett	99	2.66
15	TPC‐T28, PC‐Y2, TPC‐T29	*S. epidermidis* strain SR1	99	4.00
16	TPC‐T40	*S. aureus* subsp. *aureus* T0131	99	1.33
17	(i) TPC‐T14(S), TPC‐T33, TPC‐T25, TPC‐T9, TPC‐T39, TPC‐T49, TPC‐T15(S),	(i) *S. kloosii* strain ATCC 43959	(i) 98	9.33
	(ii) PC‐T13, TPC‐Y6, TPC‐T21, TPC‐Y4	(ii) *S. epidermidis* strain MB	(ii) 99	5.33
18	TPC‐Y1, PC‐Y10, TPC‐Y11	*S. haemolyticus* JCSC 1435	99	4.00
19	TPC‐T53, TPC‐T54	*S. hominis* strain GPL6	99	2.66
20	TPC‐T45, TPC‐T48, PC‐T8	*S. warneri* strain G72	99	
21	TPC‐T52	*K. oxytoca* strain ChDC	99	1.33
22	TPC‐T38, PC‐T4	*P. stutzeri* strain G45	99	2.66
23	PC‐Y9	*D. barathii* strain MT2.1	98	1.33

In addition, seven bacterial contaminant groups, *Bacillus*,* Corynebacterium*,* Dermacoccus*,* Enterobacter*,* Klebsiella*,* Pseudomonas*, and *Staphylococcus* were detected from the puff pastry production line with similar values exceeding 90%, except for the species *E. homaechei*. The highest abundance of bacterial contaminants was shown by *E. asburiae* (gram‐negative rods), while the lowest ones contained both gram‐positive and gram‐negative bacteria. They were *E. homaechei* (gram‐negative rods), *S. aureus* subsp. *aureus* (gram‐positive, aerobic cocci), *D. barathii* (gram‐positive coccus), and *K. oxytoca* (lactose‐fermenting, aerobic, gram‐negative rods). Interestingly, only *C. striatum* detected in the puff pastry production line, had never been previously reported.

### 3.3 Detection frequency of bacterial contaminants

Each bacterial contaminant was collected from the three different production lines, the dough‐forming line (DL), the layer‐forming‐and‐filling line (LFL), and the shock‐frozen line (SL). The detection frequencies were determined for each genus (DFG) and species (DFS) (Table 5). Among the seven genera detected, *Bacillus*,* Enterobacter*, and *Staphylococcus* exhibited high DFG of 24.00%–37.33% while the rest showed only 1.33%–2.66%. For the detection frequency at the species level *B. cereus*,* E. asburiae*,* S. kloosii*, and *S. warneri* were detected from all three lines. Their contaminations were maintained at room temperature for the first two lines (DL and LFL), and at low temperature for SL. Only five species *B. cereus*,* E. asburiae, S. kloosii*,* S. epidermidis*, and *S. warneri* were detected in the dough‐forming step. All species except *B. subtilis*,* C. striatum*, and *D. barathii* were detected at the LFL. However, many species including *B. thuringeninsis*,* E. cloacae*,* E. hormaechei*,* K. oxytoca*,* P. stutzeri*, and *S. epidermidis* could not survive at the low temperature of the SL, while three species *B. pumilus*,* S. haemolyticus*, and *S. hominis* contaminated at the LFL were tolerant at the low temperature of the SL. However, four species *B. subtilis*,* C. striatum*,* D. barathri*, and *S. aureus* only appeared at the last severe temperature step of SL. Based on detection frequency of these three lines, *E. asburiae* showed the highest DFS of 34.61%–37.5% for the first two lines, while *B. subtilis* exhibited the highest DFS of 20% for the frozen line.

**Table 5:** Detection frequency of each bacterial contaminant from the three production lines

Bacterial genus	Bacterial species	DFS (%) in each puff pastry production line			Abundance of isolates	DFG (%)
		DL	LFL	SL		
*Bacillus*	*B. cereus*	12.5	19.23	6.66	12	24.00
	*B. subtilis*	0	0	20.0	3	
	*B. thuringiensis*	0	1.92	0	1	
	*B. pumilus*	0	1.92	6.66	2	
*Enterobacter*	*E. asburiae*	37.5	34.61	6.66	22	37.33
	*E. cloacae* subsp. *cloacae*	0	9.61	0	5	
	*E. hormaechei*	0	1.92	0	1	
*Staphylococcus*	*S. kloosii*	12.5	9.61	6.66	7	30.66
	*S. haemolyticus*	0	1.92	13.33	3	
	*S. epidermidis*	25.0	9.61	0	7	
	*S. hominis*	0	1.92	6.66	2	
	*S. warneri*	12.5	1.92	6.66	3	
	*S. aureus*	0	0	6.66	1	
*Dermacoccus*	*D. barathri*	0	0	6.66	1	1.33
*Klebsiella*	*K. oxytoca*	0	1.92	0	1	1.33
*Pseudomonas*	*P. stutzeri*	0	3.84	0	2	2.66
*Corynebacterium*	*C. striatum*	0	0	13.33	2	2.66
Total		100%	100%	100%	75	100%

DL, Dough‐forming line; LFL, Layer‐forming and filling line; SL, Shock‐frozen line; DFG, detection frequency calculated as the percentage in each genus of the total isolates. DFS, detection frequency calculated as the percentage of total isolates in each species for each production line.

### 3.4 Inhibition spectra of potential AMSs against bacterial contaminants

**Figure 2:**
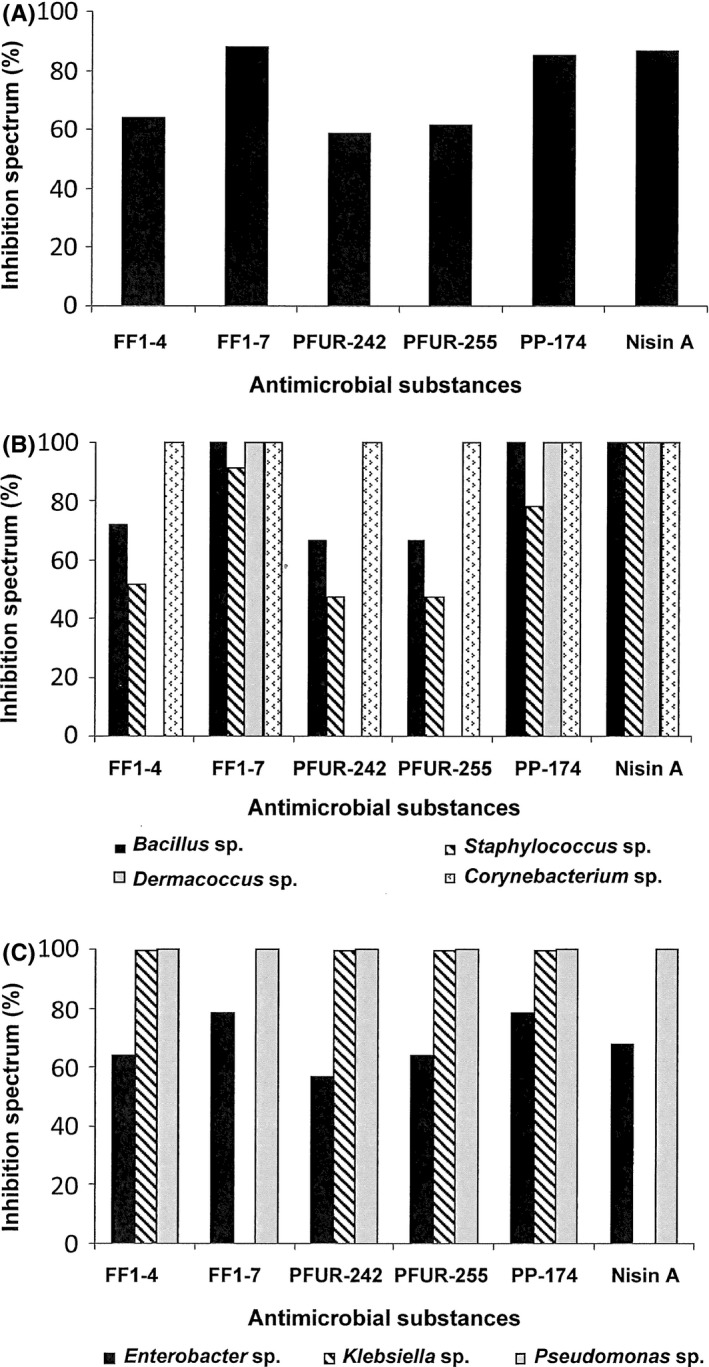
Inhibition spectra of six AMSs from lactic acid bacteria and the commercial bacteriocin nisin A in bacterial contaminated puff pastry: (A) inhibition spectra against all bacterial contaminants by six AMSs (FF1‐4, FF1‐7, PFUR‐242, PFUR‐255, PP‐174, and nisin A); (B) inhibition spectra of AMSs against gram‐positive bacteria; and (C) inhibition spectra of AMSs against gram‐negative bacteria

Six antimicrobial substances FF1‐4, FF1‐7, PFUR‐242, PFUR‐255, PP‐174, and nisin A were tested for their inhibition spectra against all bacterial contaminants in puff pastry production, resulting in 64.00%, 88.00%, 58.67%, 61.33%, 85.33%, and 86.66%, respectively (Fig. 2A). Only nisin A, FF1‐7, and PP‐174 exhibited high inhibition spectra of 78–100% against all gram‐positive bacteria (Fig. 2B) while 0.02 N HCl buffer used as a negative control resulted no activity. All except nisin A and FF1‐7 exhibited 100% inhibition spectra against *Klebsiella*, and showed inhibition activities of 57%–58% against *Enterobacter* (Fig. 2C). Nisin A and FF1‐7 did not inhibit the growth of *Pseudomonas* sp*. *Nisin A and PP‐174 displayed effective inhibition against 67 and 64 of the isolates studied, respectively. However, 21 resistant isolates of *E. asburiae*,* E. cloacae*,* E. homaechei*,* K. oxytoca*,* S. epidermidis*,* S. kloosii*, and *S. warneri* were not inhibited by either nisin A or PP‐174 (Table 2). These resistant isolates were further tested for possible inhibition by a combination of nisin A and PP‐174.

### 3.5 Inhibition activity of antimicrobial substances PP‐174, nisin A and the combination of PP‐174 and nisin A, against adhering bacterial cells to conveyor belt coupons

Bakery products are now a convenience food source suiting the lifestyles of Thai people. They are generally considered as microbiologically safe due to the high temperatures (up to 170°C) used in the baking process. However, the conveyor belts used for transfer, either pre‐ or postbaking are the most likely sources of bacterial contamination. The efficiency of AMSs, including PP‐174 and nisin A alone, as well as in combination against the 21 resistant bacterial contaminants was investigated using a conveyor model system. The bacterial quantification of the conveyor belt coupons used in this experiment, showed that both PP‐174 and nisin A alone significantly reduced the growth of mixed target isolates from approximately 5.6–5.8 log CFU/coupon to <1 log CFU/coupon at 4 and 12 hr, respectively, (*p *≤* *.05). The combination of PP‐174 and nisin A reduced growth to <1 log CFU/coupon at 0 hr (Table 6).

**Table 6:** Inhibition of the antimicrobial substances PP‐174 and nisin A, alone and in combination against mixed 21 target isolates on conveyor belt coupon

Treatment	Survival cells (logCFU/coupon)				
	0 hr	4 hr	8 hr	12 hr	16 hr
Control	5.81 ± 0.06^aA^	5.67 ± 0.08^aA^	5.62 ± 0.11^aA^	5.65 ± 0.30^aA^	5.6 ± 0.07^aA^
PP‐174	3.24 ± 0.33^bA^	<1	<1	<1	<1
Nisin A	3.65 ± 0.06^bA^	3.39 ± 0.12^bAB^	3.15 ± 0.21^bB^	<1	<1
PP‐174 and nisin A	<1	<1	<1	<1	<1

^a,b,A^Values are mean ± standard deviations of one‐way ANOVA determinations performed in duplicate. Means with lowercase and uppercase letters within the same column and row, respectively, are significantly different (*p *<* *.05) determined using.

## 4 Discussion

To investigate high efficiency of AMSs for a tropical region like Thailand, the microbial contaminants of a commercial bakery production were collected. Seventy‐five resistant bacterial contaminants were isolated from each compartment and grouped using RAPD analysis. Based on 16S rDNA sequence analysis, seven genus of bacterial contaminants were successfully identified. Usually, *Bacillus* species are proposed as bacterial contaminants in food production. They can be found during food preparation and processing, transportation and storage, or even in food handling by the worker (Bennett, Walsh, & Gould, 2013). In this study, *B. cereus* was detected with high DFS compared to other Bacilli species along the three production lines. *B. cereus* usually occurs in flour and other raw materials. Its spores are resistant to heat desiccation and temperatures as low as −20°C, and can commence growth from 10*°*C (Blackburn, 2006; Rosenkvist & Hansen, 1995; Seiler, 1984). In addition, it can adhere to equipment surfaces and pipelines because of the hydrophobic character of the exosporium, and the presence of appendages on the spore surface (Heyndrickx, 2011). These characters explain why it was detected at high DFS compared to the other Bacilli species. Both *B. subtilis* and *B. pumilus* also appeared in the shock‐frozen line, indicating that these two species can also tolerate low temperature. Therefore, contamination by these three Bacilli species at low temperature could be used as indicators for finish products regarding food safety considerations.

Among the six species of Staphylococci found, three *S. aureus*,* S. epidermidis*, and *S. haemolyticus* can produce extracellular staphylococcal enterotoxins (Lawley, Curtis, & Davis, 2008; Lindsay & Holden, 2004; Omoe et al., 2005). Only two species, *S. aureus* and *S. haemolyticus* were detected at the final step of the frozen line, whereas *S. epidermidis* disappeared. However, its toxin may still remain in the finish product and this should be considered for food safety. Staphylococci are normally found in the air, dust, water, food, humans and animals, and on environmental surfaces (Hait, Tallent, Melka, Keys, & Bennett, 2014). Staphylococci sp. also result from protein sources such as fresh beef, soybean curd, shells, and the yolk of quail eggs used to prepare fillings (Ananchaipattana et al., 2012; Goja, Ahmed, Saeed, & Dirar, 2013; Pyzik & Marek, 2012). Therefore, special care should be taken of these raw materials with strong control procedures.

Not only gram‐positive bacteria were found, the gram‐negative bacteria *Enterobacter* was also detected at the highest DFG of 37.33%. Three species *E. asburiae, E. cloacae* subsp. *Cloacae*, and *E. hormaechei* were found in the production process. They are often found in soil, water, and plants (Asis & Adachi, 2004; Lau, Sulaiman, Chen, Yin, & Chan, 2013), and can live as normal flora in the gastrointestinal tract (Koth, Boniface, Chance, & Hanes, 2012). Among of these, *E. hormaechei* can be isolated from blood, wounds, or sputum. In addition, it is a pathogen causing nosocomial infections including sepsis (Townsend, Hurrell, Caubilla‐Barron, Loc‐Carrillo, & Forsythe, 2008). Only *E. asburiae* existed in all three production lines, the other two species disappeared in the shock‐frozen line. *E. asburiae* survived in the shock‐frozen line, subjected to a temperature of −60°C to −70°C for 13–18 min. Normally, the optimum temperature for growth of *E. asburiae* is 30°C (Garrity, Brenner, Krieg, & Staley, 2007), but no previous studies reported the survival of *E. asburiae* at temperatures below 0°C. Our results indicated that this species can survive at subzero temperatures. *E. asburiae* also displayed the highest abundance at 29% compared to the other species. Therefore, it can be used as another biomarker to indicate the quality of finish products, especially frozen puff pastry.

Low detection frequencies were displayed by *D. barathri*,* K. oxytoca, P. stutzeri*, and *C. striatum*. Both *K. oxytoca* and *P. stutzeri* appeared in the second line (LFL), but disappeared in the low‐temperature environment. This can be explained by their growth temperature of 4–45°C (Brisse, Grimont, & Grimont, 2006; Lalucat, Bennasar, Bosch, García‐Valdés, & Palleroni, 2006; Pathom‐aree et al., 2006; Wilson, 2005). However, Both *D. barathri* and *C. striatum* appeared in the last line (SL), indicating that they could survive in low temperatures of −60°C to −70°C for 13–18 min. *C. striatum* is a common skin flora (Buchta et al., 2004) and can be isolated from humans (Martínez‐Martínez, Suárez, Rodríguez‐Baño, Bernard, & Muniáin, 1997), or indoor air (Li et al., 2014). However, *C. striatum* has not been previously reported in bakery products. It is a nosocomial pathogen, with infection sites of the blood stream, lung, and central nervous system (Lee, Ferguson, & Sarubbi, 2005). Possible contamination might be via workers and air. This is a serious issue to consider in the future. Another contaminant species, *K. oxytoca* and *P. stutzeri* are also an opportunistic pathogen in hospital or clinical setting (Lowe et al., 2012; Noble & Overman, 1994). Additionally, *D. barathri* causes catheter‐related blood stream infection (CRBSI) in human (Takahashi et al., 2015). Therefore, these strains should be concerned for food production.

Recently, several unit operations and treatments used during food processing such as drying, cold storage, modified atmosphere storage, heat treatment, and chemical preservatives have been applied to extend the shelf life of food products (Reis, Paula, Casarotti, & Penna, 2012; Schnürer & Magnusson, 2005). However, they may exert undesirable effects on the texture and flavor of the food. Thus, biopreservation, especially AMS produced by LAB has become more interesting. Investigation of AMS produced by LAB in this study to inhibit the growth of those resistant bacterial contaminants after cleaning system applied resulted that the combination of PP‐174 and nisin A at the concentration of 500 AU/ml each was successful to completely inhibit the growth of those contaminants at 0 hr and they were not detected up to 16 hr. Amin (2012) and Perez‐Perez, Regalado‐González, Rodríguez‐Rodríguez, Barbosa‐Rodríguez, and Villaseñor‐Ortega (2006) proposed that no single antimicrobial agent covered all the requirements for food preservation. Nisin is a well‐known bacteriocin which is declared to be GRAS (generally recognized as safe) and used in combination with other antimicrobial compounds (Siroli et al., 2016). Nisin can bind to lipid II, the main transporter of peptidoglycan subunits from the cytoplasm to the cell wall, resulting in the prevention of proper cell wall synthesis, leading to cell death due to small intracellular metabolites released. Furthermore, lipid II was used as a docking molecule to initiate a process of membrane insertion and pore formation causing rapid cell death (Cotter, Hill, & Ross, 2005; Wiedemann et al., 2001). The mixture of different bacteriocin including lactocin 705 (17,000 AU/ml), enterocin CRL35 (17,000 AU/ml), and nisin A (2,000 IU/ml) inhibited 100% gram‐positive bacteria *Listeria monocytogenes* and *L. innocua* (10^6^ CFU/ml) in a broth and meat system after 24 hr of incubation (Vignolo et al., 2000). In addition, Twele et al. (2011) proposed the formulation comprising three inactivated culture solution of *Carnobacterium maltaromaticum *ATCC^®^ PTA‐9380, *C. maltaromaticum *ATCC^®^ PTA‐9381, and *Enterococcus mundtii *ATCC^®^ PTA‐9382 as well as nisin at a concentration ranging from 500 to 5,000 IU/ml used as a sanitizer to inhibit the pathogen *Listeria monocytogenes* in the food industry. It seemed that the combination of various bacteriocins successfully inhibited those gram‐positive bacteria. However, many studies reported for the combination of bacteriocins and other chemical compounds to inhibit the growth of Gram‐negative bacteria. A combination of nisin A at 500, 800, or 1,000 IU/ml and 20% ethanol successfully inhibited the growth of gram‐negative bacteria including *E.coli* O157:H7, *Salmonella* Typhimurium TISTR 292, and *S. *Enteritidis DMST 17368 to below 1 log CFU/ml at 15 min. The minimum inhibitory concentration of nisin A and ethanol was 500 IU/ml and 20% (v/v), respectively (Phongphakdee & Nitisinprasert, 2015). While a mixture of 25 μg/ml enterocin AS‐48 and 0.1–2.0% polyphosphoric acid partially reduced or inhibited the growth in soybean sprouts of *Salmonella enterica*,* E. coli* O157:H7, *Enterobacter aerogenes*,* Yersinia enterocolitica*,* Aeromonas hydrophila*, and *P. fluorescens* to at least 2 log when stored at 15°C (Molinos et al., 2008). Wongsuttichote and Nitisinprasert (2009) also found that 0.5% partial purified bacteriocin PP‐174 displayed 100% growth inhibition activities against aerobic mesophiles and Gram‐negative bacteria coliform, but not to LAB. Additional low concentration of 17.42 mmol/L lactic acid and 6.91 mmol/L acetic acid to 0.5% PP‐174 to form food preservative BAKE‐SAFE‐1 could completely inhibit the growth of LAB. In this study, it was successfully to form a combination of two bacteriocins, PP‐174 and nisin A to inhibit both gram‐positive and gram‐negative bacteria which can be used as a disinfectant agent for food industry in the future.

## 5 Conclusions

Seven genera of bacterial contaminants *Bacillus*,* Corynebacterium*,* Dermacoccus*,* Enterobacter*,* Klebsiella*,* Pseudomonas*, and *Staphylococcus* were successfully identified by 16SrDNA sequence analysis. Effective AMSs PP*‐*174 and nisin A exhibited the growth inhibition of different target bacterial contaminants from puff pastry industry. However, their combination of PP‐174 and nisin A provided 100% inhibition spectra against all those food spoilage and pathogenic bacteria, and can be used as a potential disinfectant agent for food industry in the future.

## Funding Information

No funding information provided.

## Conflict of Interest

None declared.
